# Low temperature synthesis via molten-salt method of r-BN nanoflakes, and their properties

**DOI:** 10.1038/s41598-019-52788-0

**Published:** 2019-11-08

**Authors:** Yang Chen, Xing Wang, Chao Yu, Jun Ding, Chengji Deng, Hongxi Zhu

**Affiliations:** The State Key Laboratory of Refractories and Metallurgy, Wuhan University of Science and Technology, Wuhan, 430081 China

**Keywords:** Structural properties, Synthesis and processing

## Abstract

r-BN nanoflakes were synthesized using KBH_4_ and NH_4_Cl as the main raw material in a high-purity nitrogen atmosphere. The effects of salt and salt-free conditions and heating temperature on the synthesis of BN were studied. The molten-salt method was used to synthesize BN at 650 °C, which was 250 °C lower than the BN synthesis method without salt. Furthermore, at 1000 °C the prepared flake-like BN crystals showed good crystallinity, uniform morphology, a particle diameter of 200–300 nm, and a thickness of 40–70 nm. Moreover, the specific surface area of BN was 294.26 m^2^/g. In addition, the BN synthesized at 1100 °C had a large elastic modulus value and good oxidation resistance.

## Introduction

Boron nitride (BN) is a new type of ceramic material with excellent performance and great potential for development. Due to its low dielectric constant^1^, wave transparency^2,3^, good electrical insulation^4^, low thermal expansion^5^, high thermal conductivity^6^, high-temperature lubricity^7^, high-temperature stability^8^, and wide band gap^9^. Common BN contains hexagonal boron nitride (h-BN) with a graphite layered structure, cubic boron nitride (c-BN) with a diamond sphalerite structure, wurtzite boron nitride (w-BN) with a hexagonal diamond wurtzite structure, rhombohedral boron nitride (r-BN) with a trigonal phase graphite structure, turbostratic boron nitride (t-BN) with a laminar structure, and amorphous boron nitride^10–15^. Of these, r-BN has high chemical stability and oxidation resistance, which makes it suitable for high-temperature resistant materials. Moreover, it also has a wide band gap, high thermal conductivity, and high resistivity, and can be used as an ideal substrate and heat sink material for high-power, high-temperature, and semiconductor materials.

At present, the research on BN is mainly focused on h-BN^16–20^ and c-BN^21–23^, and there has been little research on r-BN. In recent years, researchers have mainly used chemical vapor deposition (CVD) to synthesize r-BN. Chubarov *et al*. performed an in-depth study of r-BN. They used triethylboron and ammonia as precursors, and via CVD, r-BN was produced on a sapphire substrate at 1500 °C and a pressure of 7 kPa. The addition of a small amount of SiH_4_ to the gas mixture was also studied, and it was found that Si atoms acted as surface-active substances that stimulated the formation of high-quality r-BN^24^. Subsequently, they formed an AlN buffer layer by *in-situ* nitridation, and BN was grown under low pressure using H_2_ as a carrier gas at 1200–1500 °C^25^. After SiC was used as the substrate, it was found that growth temperature was 1500 °C, the N / B ratio was 642 and the deposition pressure was 7 kPa favored r-BN epitaxial growth, and no buffer layer was required^26^. In addition, Oku *et al*.^27^ prepared r-BN particles of 50–1000 nm diameter via CVD on a graphite substrate from a BCl_3_-NH_3_-H_2_ reaction system at 1600 °C and a total pressure of 3–5 Torr. Most of these methods require a high reaction temperature, a certain pressure, or a particular explosive atmosphere, and the purity of the synthesized r-BN is not high.

Other methods have also been used to prepare r-BN. Bao *et al*.^28^ prepared highly crystalline r-BN triangular nanosheets by using NaNH_2_ and B_2_O_3_ as raw materials in a solid-phase reaction in an autoclave at 600 °C for 6 h. The obtained r-BN triangular nanosheets had a width of approximately 300–500 nm and a thickness of approximately 50–90 nm, but this method required a long reaction time and a certain pressure. Ye *et al*.^13^ prepared r-BN powders by using Na_2_B_4_O_7_ and Mg as raw materials at 1000 °C for 3 h under a nitrogen atmosphere with NaCl molten salt. The prepared BN was not high in purity and it contained h-BN, and the reaction temperature was high. Therefore, further research is still needed to improve the production process of r-BN to obtain an r-BN preparation method that is simple and safe, uses a low synthesis temperature, and produces high yield.

In this work, we report on r-BN synthesized in a NaCl-KCl eutectic salt and a nitrogen atmosphere by using KBH_4_ and NH_4_Cl as the main raw materials, and compare the effect of the salt-free condition on the synthesis of r-BN. High-purity r-BN was synthesized at 650 °C and normal pressure by using the molten-salt synthesis (MSS)^29–34^ method, which was 250 °C lower than the temperature required when no salt was added, and it needed a shorter reaction time. The effect of reaction temperature on the phase and microstructure of r-BN was studied. In addition, the chemical valence, optical properties, and specific surface area of the as-prepared r-BN nanoflakes were also investigated. One possible formation mechanism of r-BN is put forward. Finally, the prepared r-BN nanoflakes were subjected to antioxidant analysis.

## Methods

### Preparation of r-BN nanoflakes

Potassium borohydride (KBH_4_, 97.0% purity, Shanghai Aladdin Biochemical Technology Co., Ltd.) and ammonium chloride (NH_4_Cl, 99.5% purity, Tianjin Kaitong Chemical Reagent Co., Ltd.) were used as the main raw materials; sodium chloride (NaCl, 99.5% purity, Shanghai Aladdin Biochemical Technology Co., Ltd.) and potassium chloride (KCl, 99.5% purity, Shanghai Aladdin Biochemical Technology Co., Ltd.) were used as the molten salt to synthesize r-BN in high-purity nitrogen (99.999%). The raw materials (KBH_4_/NH_4_Cl, molar ratio of 1:2) and molten salt (molar ratio of NaCl/KCl = 1:1) were mixed at a mass ratio of 6:5. The mixture was heated from 25 °C at a ramp of 2 °C/min to 300 °C in a vertical corundum tube furnace, and then heated to 650–1100 °C at a rate of 4 °C/min and held for 4 h. After that, the reaction products were cooled in the furnace to room temperature. The reaction products were soaked in distilled water for 2 h and washed with distilled water two to three times by using an ultrasonic cleaner to remove unreacted materials and molten salts. The final product was then dried at 110 °C to obtain r-BN nanoflakes.

Because NH_4_Cl would become volatile above 330 °C, the mixture was heated from 25 °C to 300 °C at a rate of 2 °C/min so that KBH_4_ and NH_4_Cl could produce more stable substances to reduce the loss in the subsequent NH_4_Cl heating process, and the amount of NH_4_Cl was greater than that of KBH_4_ to compensate for the loss of NH_4_Cl at high temperature.

The reaction for synthesizing BN can be described as follows:

1 $${{\rm{KBH}}}_{4({\rm{s}})}+{{\rm{NH}}}_{{\rm{4}}}{{\rm{Cl}}}_{({\rm{s}})}\to {{\rm{BN}}}_{({\rm{s}})}+{{\rm{KCl}}}_{({\rm{s}})}+{{\rm{4H}}}_{{\rm{2}}({\rm{g}})}$$

$$\Delta {{\rm{G}}}^{{\rm{\theta }}}=-\,265.01-0.41{\rm{T}}\,({\rm{KJ}}\cdot {{\rm{mol}}}^{-1})$$

### Characterization

The phases of the products were analyzed by X-ray diffraction (XRD, PANalytical, X’Pert Pro) using CuKα radiation. The element species and chemical valences of the samples’ surfaces were studied by X-ray photoelectron spectroscopy (XPS, Thermo Fisher Scientific, ESCALAB 250Xi) with AlKα radiation. The morphology and crystal structure were observed by scanning electron microscopy (SEM, FEI, Nova 400 Nano SEM), high-resolution transmission electron microscopy (HRTEM, JEOL, JEM-2100 UHR STEM/EDS) and atomic force microscopy (AFM, NT-MDT Prima). In addition, a synchronous thermal analyzer with thermogravimetric analysis-differential thermal analysis (TG-DTA, NETZSCH, STA449F3) was used to investigate the oxidation resistance of the as-prepared BN, and the generation process of the BN. The infrared absorption of the product was tested using the Fourier transform infrared (FTIR, Thermo Fisher Scientific, Nicolet iS 50) spectrum. The Brunauer–Emmett–Teller (BET) specific surface area of the as-prepared BN was characterized using a fully automatic surface area and porosity analyzer (JWGB, JW-BK100C).

## Results and Discussion

### Phase analysis

**Figure 1:**
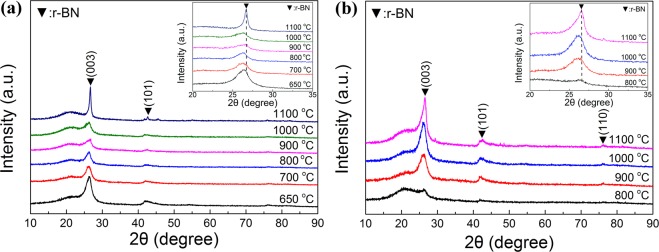
XRD patterns of samples heated at different temperatures for 4 h: (**a**) With salt; (**b**) Without salt.

Figure 1a shows the XRD patterns of samples heated at different temperatures for 4 h in NaCl-KCl eutectic salts. At 650 °C, the diffraction peak of BN appeared at 25–30°, but the amount of BN obtained after water washing of the fired product was small. This is because the molten salt partially melted (the eutectic melting temperature of NaCl-KCl is 657 °C), and generated a small amount of liquid phase environment. The raw materials of KBH_4_ and NH_4_Cl were mostly evaporated or decomposed after a long time at low temperature. This indicates that an excessive holding time at low temperatures was not conducive to the synthesis of BN. As the heating temperature rose to 1100 °C, the main diffraction peak of BN became sharp, and the peak intensity was maximized. This shows that BN synthesized at 1100 °C had the best crystallinity. Furthermore, there are two diffraction peaks: the peaks at diffraction angles (2θ) of 26.717° and 42.620° can be indexed to r-BN ((003) and (101)), respectively. In addition, a small amount of O_2_ from the N_2_ gas caused slight oxidation of the BN during the synthesis. A weaker hump is found at a diffraction angle of 22°, which corresponds to amorphous B_2_O_3_.

Figure 1b shows the XRD patterns of samples heated at different temperatures for 4 h without adding salt. No BN was generated at 800 °C. When the heating temperature was 900 °C, BN started to form. At 900–1100 °C, as the heating temperature increased, the peak intensity of BN increased and its peak became sharper. However, as the heating temperature rose to 1100 °C, the diffraction peak of the (003) crystal plane showed a slight shift in the higher angle direction, accompanied by a narrowing of the diffraction peak. Figure 1a also shows this phenomenon. According to the Bragg equation ($2{\rm{d}}\,\sin \,\theta ={\rm{n}}\lambda$), the diffraction peak is shifted to a larger angle, the lattice spacing of BN is decreased, and the lattice is shrunken along the c-axis orientation. The grain size is larger in the direction perpendicular to the (00 *l*) direction, resulting in a narrowing of the (003) peak by continuous X-ray scattering^35^. In addition, a small diffraction peak appears at an angle of 75.955°, which is the (110) crystal plane of r-BN.

**Figure 2:**
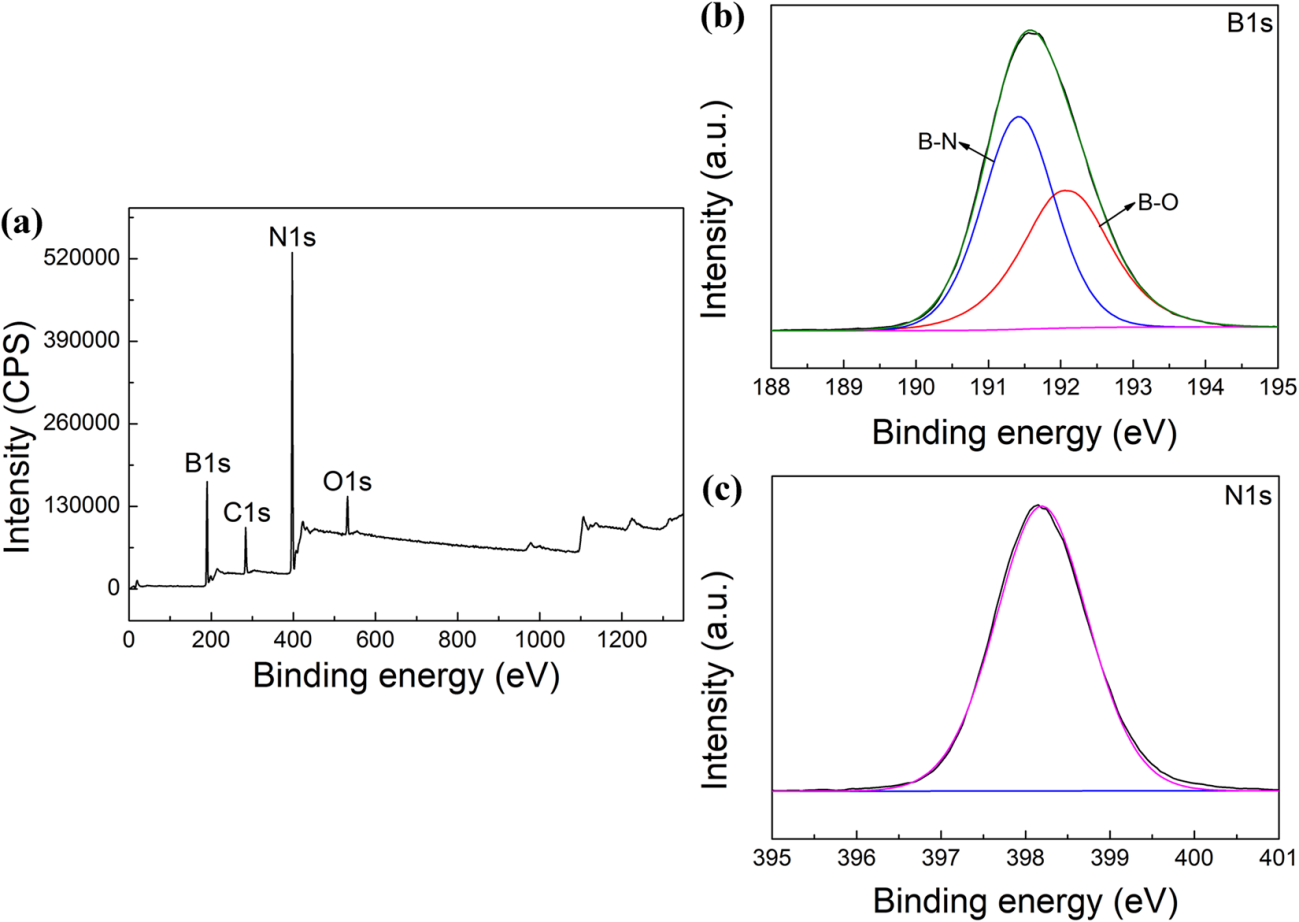
XPS spectra of BN synthesized at 1100 °C in NaCl-KCl eutectic salt: (**a**) The full spectrum of the product XPS; (**b**) B1s spectra of BN; (**c**) N1s spectra of BN.

XPS can be used to check all elemental information in a substance. Figure 2a shows the full spectrum of the product XPS. In Fig. 1a, the O1s peak (532.77 eV) is attributed to amorphous B_2_O_3_. The C1s peak (284.94 eV) is attributed to the addition of C as an internal standard substance. The binding energies of B1s and N1s are 190.75 eV and 398.09 eV, respectively. As shown in Fig. 2b, the B1s spectrum has two peaks at 191.42 eV and 192.04 eV, corresponding to B-N and B-O, respectively. Moreover, the N1s spectrum has a peak at 398.19 eV, corresponding to N-B^36^ (Fig. 2c). From the elemental content calculation of the XPS data (Table 1), the product consists of 91.87% BN and 8.13% B_2_O_3_.

**Table 1:** The relative content of elements in the XPS spectra of Fig. 2.

Peak	Peak BE (eV)	FWHM (eV)	Area	Atomic (%)
O1s	532.77	3.23	1.917 × 10^5^	3.46
C1s	284.94	3.04	2.098 × 10^5^	9.64
N1s	398.09	2.82	1.351 × 10^6^	39.08
B1s	190.75	2.71	4.082 × 10^5^	47.82

### Microstructure analysis

**Figure 3:**
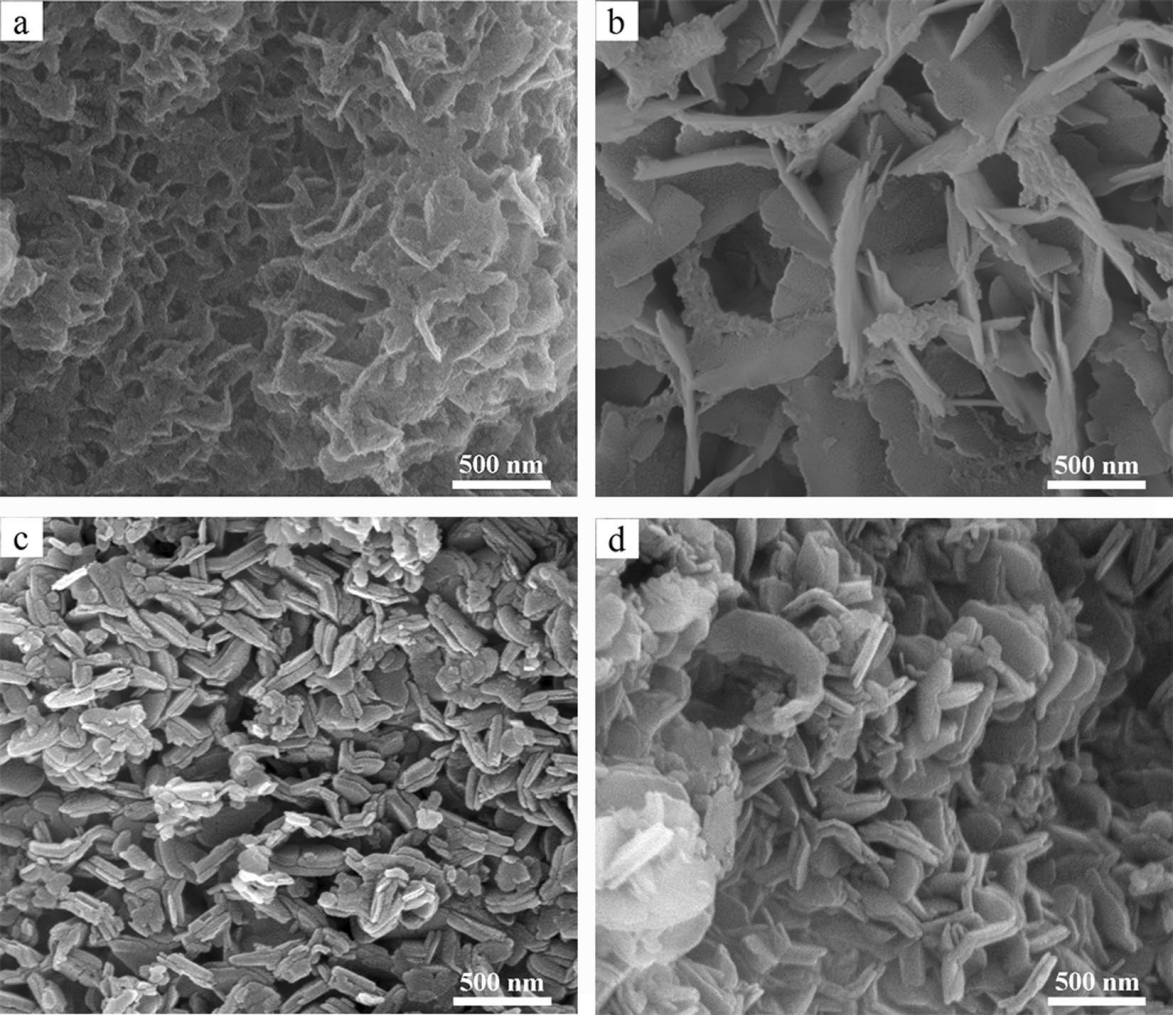
SEM images of BN synthesized at different temperatures in NaCl-KCl eutectic salts: (**a**) 800 °C; (**b**) 900 °C; (**c**) 1000 °C; (**d**) 1100 °C.

Figure 3 shows SEM images of the BN synthesized at different temperatures in NaCl-KCl eutectic salts. Figure 3a shows many thin and poorly crystallized BN nanoflakes at 800 °C, which are stacked in a staggered relation to each other to form many small holes. The production of these holes may have been caused by the volatilization of the raw materials or the gases produced by the reaction. When the heating temperature rose to 900 °C, BN nanoflakes grew (Fig. 3b). At 1000 °C, the as-synthesized BN has a uniform morphology with a diameter of 200–300 nm and a thickness of 40–70 nm, and the two grains grow close together (Fig. 3c). As the heating temperature was increased to 1100 °C, the morphology of the BN was approximately unchanged, but some BN nanoflakes became curled (Fig. 3d). This may have occurred to reduce the surface free energy, so that the BN nanoflakes were in the lowest energy state to eliminate the internal stress of the flake structure. The nanoflake diameter is approximately 300–500 nm and the thickness is approximately 25–50 nm.

**Figure 4:**
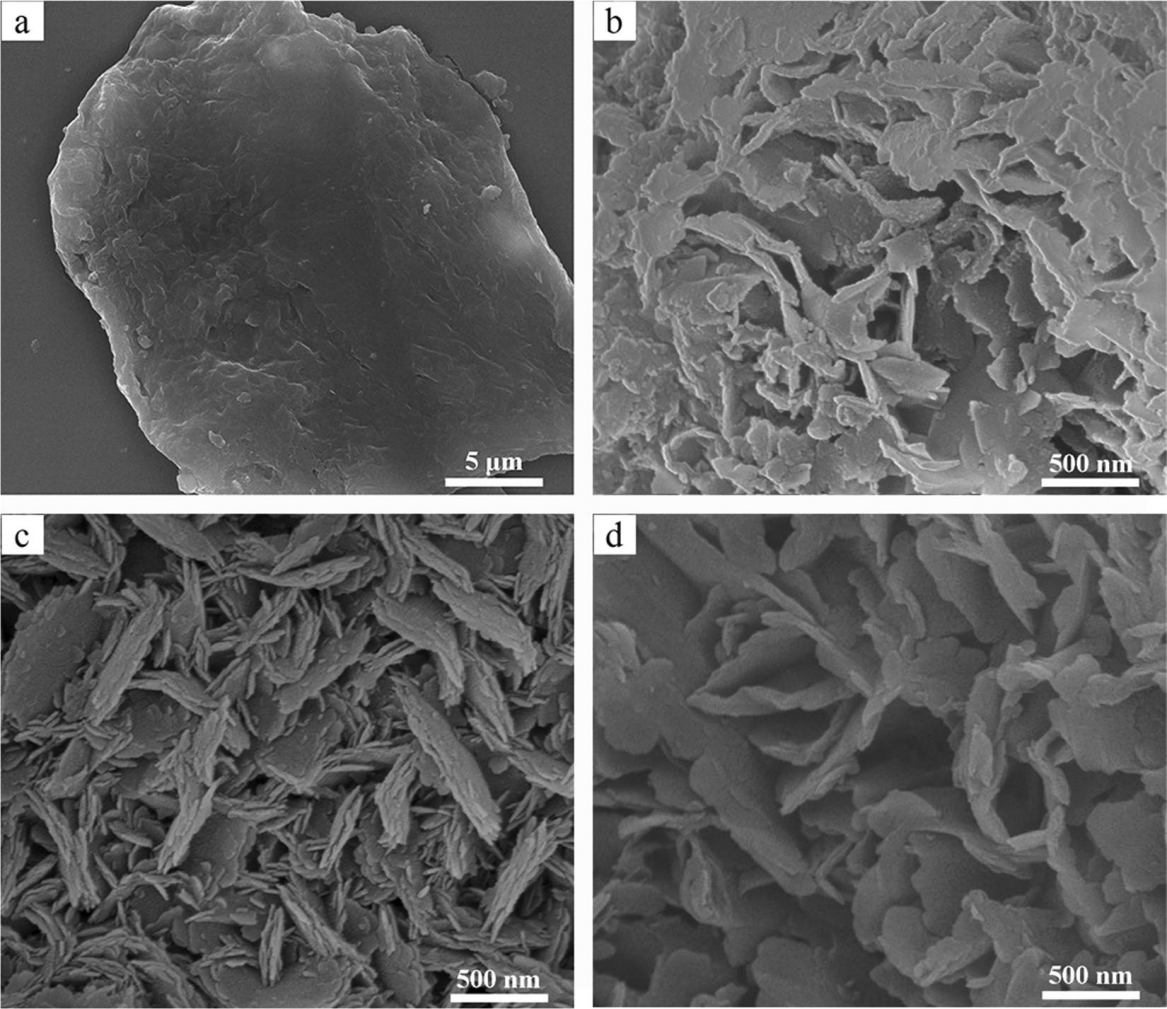
SEM images of BN synthesized at different temperatures without adding salt: (**a**) 800 °C; (**b**) 900 °C; (**c**) 1000 °C; (**d**) 1100 °C.

Figure 4 shows SEM images of the BN synthesized at different temperatures without adding salt. Figure 4a shows that irregular grains were generated at 800 °C, and the low temperature was not conducive to the growth of grains. Some irregular BN nanoflakes appear at 900 °C (Fig. 4b). When the temperature is 1000 °C, the BN nanoflakes are not uniform in size, and many small flake-like particles of BN are formed on the large flake-like BN, and the small flake-like BN has grown tightly together (Fig. 4c). As the temperature rose to 1100 °C, the flake-like BN grew to a diameter of approximately 400–700 nm, and a thickness of approximately 50–75 nm (Fig. 4d).

**Figure 5:**
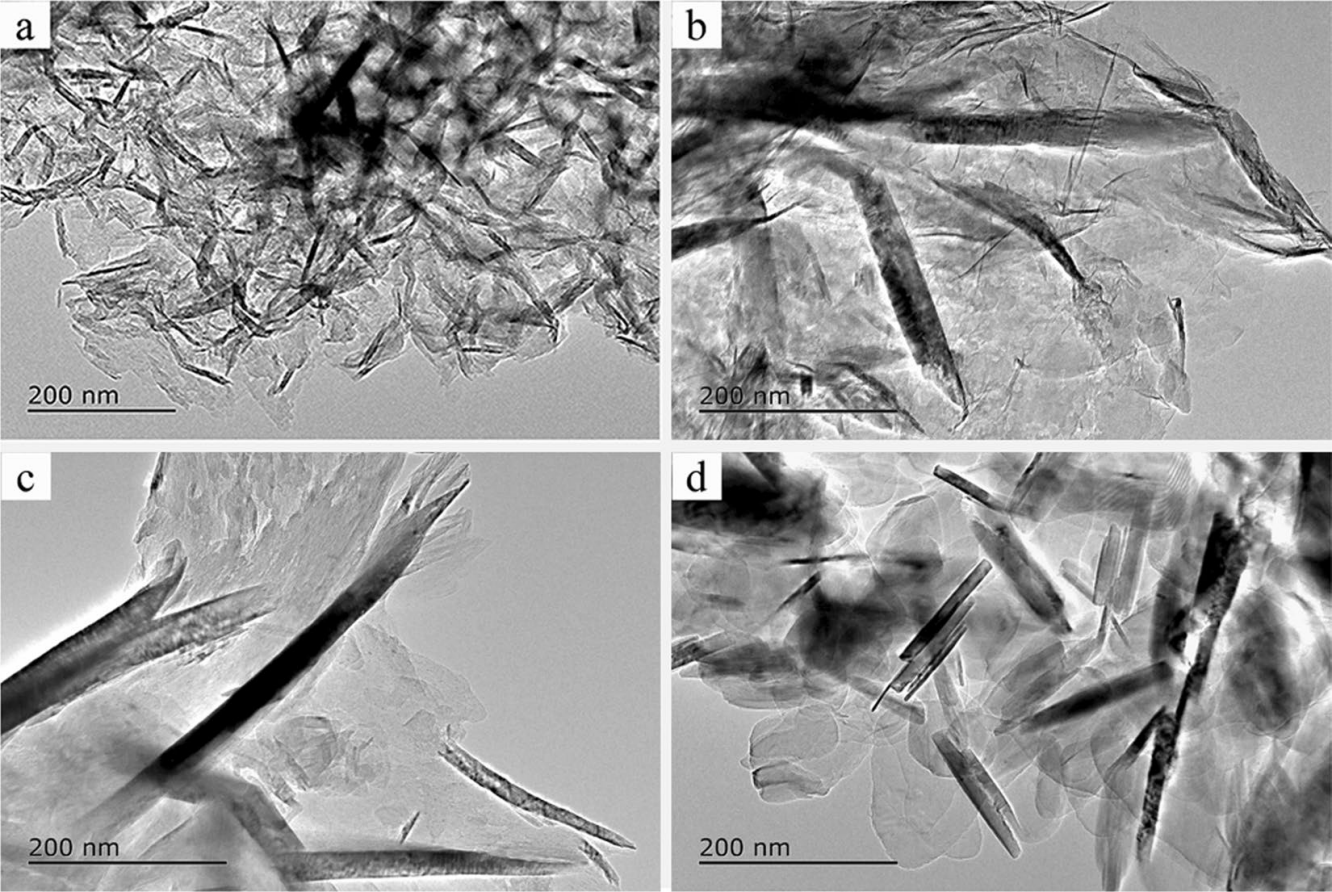
TEM images of BN synthesized at different temperatures in NaCl-KCl eutectic salts: (**a**) 700 °C; (**b**) 800 °C; (**c**) 900 °C; (**d**) 1000 °C.

The effects of different temperatures on the morphology and crystal structure of BN formed in a molten-salt environment were further investigated by TEM (Fig. 5). Figure 5a shows that the BN is very thin, and has grown with a staggered profile, with an average thickness of approximately 10 nm at 700 °C. At 800 °C, the crystallinity of the BN is not good, and the BN nanoflakes have rough boundaries and disordered edges (Fig. 5b). At 900 °C, the tip of the generated BN is clearly bifurcated, which could be described as two grains of BN grown into one grain along the c-axis direction (Fig. 5c). At 1000 °C, the BN nanoflakes have grown closely together and are of uniform size (Fig. 5d), which is consistent with Fig. 3c, and this further shows that the morphology of the BN synthesized at 1000 °C is better.

**Figure 6:**
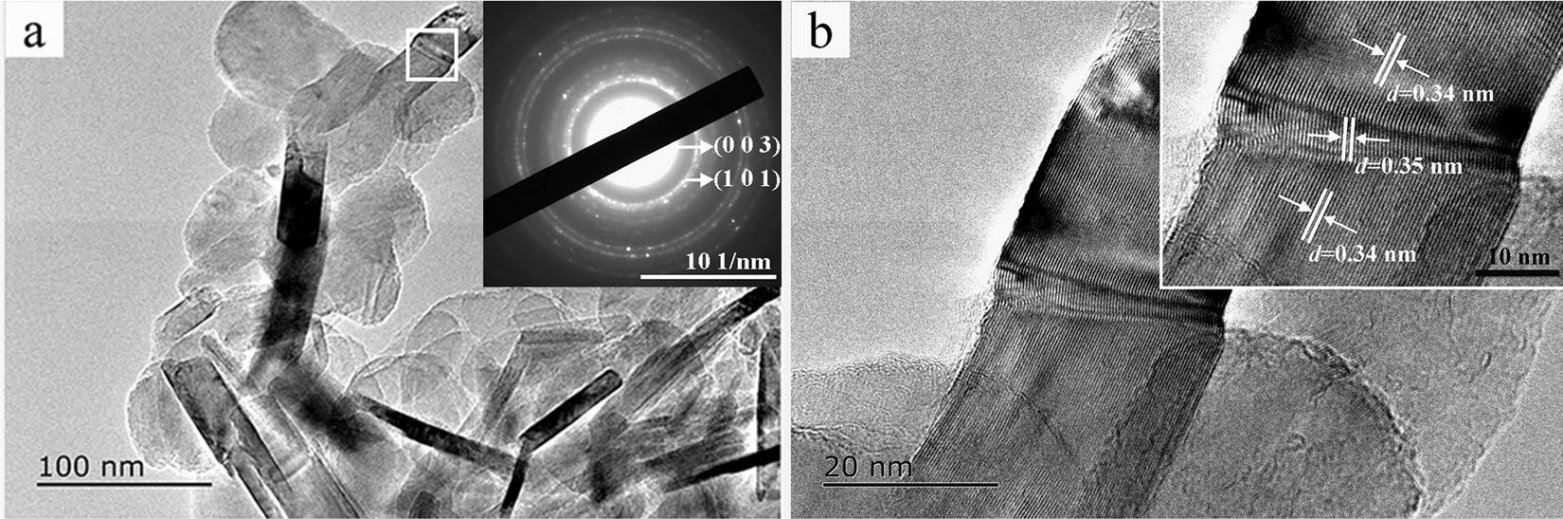
TEM and SAED (**a**), HRTEM (**b**) images of BN synthesized at 1100 °C in NaCl-KCl eutectic salts.

In addition, Fig. 6 shows the TEM, HRTEM, and selected area electron diffraction (SAED) images of BN synthesized at 1100 °C in NaCl-KCl eutectic salts. Figure 6a shows a typical TEM image of BN nanoflakes, which have a diameter of 70–130 nm and a thickness of 5–30 nm, and some BN nanoflakes are wrinkled. The inset in Fig. 6a shows the SAED pattern of r-BN with some diffraction spots distributed over the diffraction rings, indicating that the BN nanoflakes have a polycrystalline structure. The diffraction rings are indexed as the (003) and (101) planes of r-BN, which corresponds to the XRD results. Figure 6b is the HRTEM image of the BN in Fig. 6a. The lattice spacing of the BN in the smooth area is 0.34 nm, which corresponds to the (003) plane of r-BN (3.334 Å, ICDD No. 00-045-1171)^13^, indicating that the BN nanoflakes grew in a direction perpendicular to the (003) crystal plane. This directly confirms our interpretation of the phenomenon shown in Fig. 1. However, the lattice spacing at the fold is 0.35 nm. This may be due to the rotation of the lattice plane, which caused the BN nanoflakes to curl and exhibit folds on the crystal plane^20^, as in Fig. 3d.

**Figure 7:**
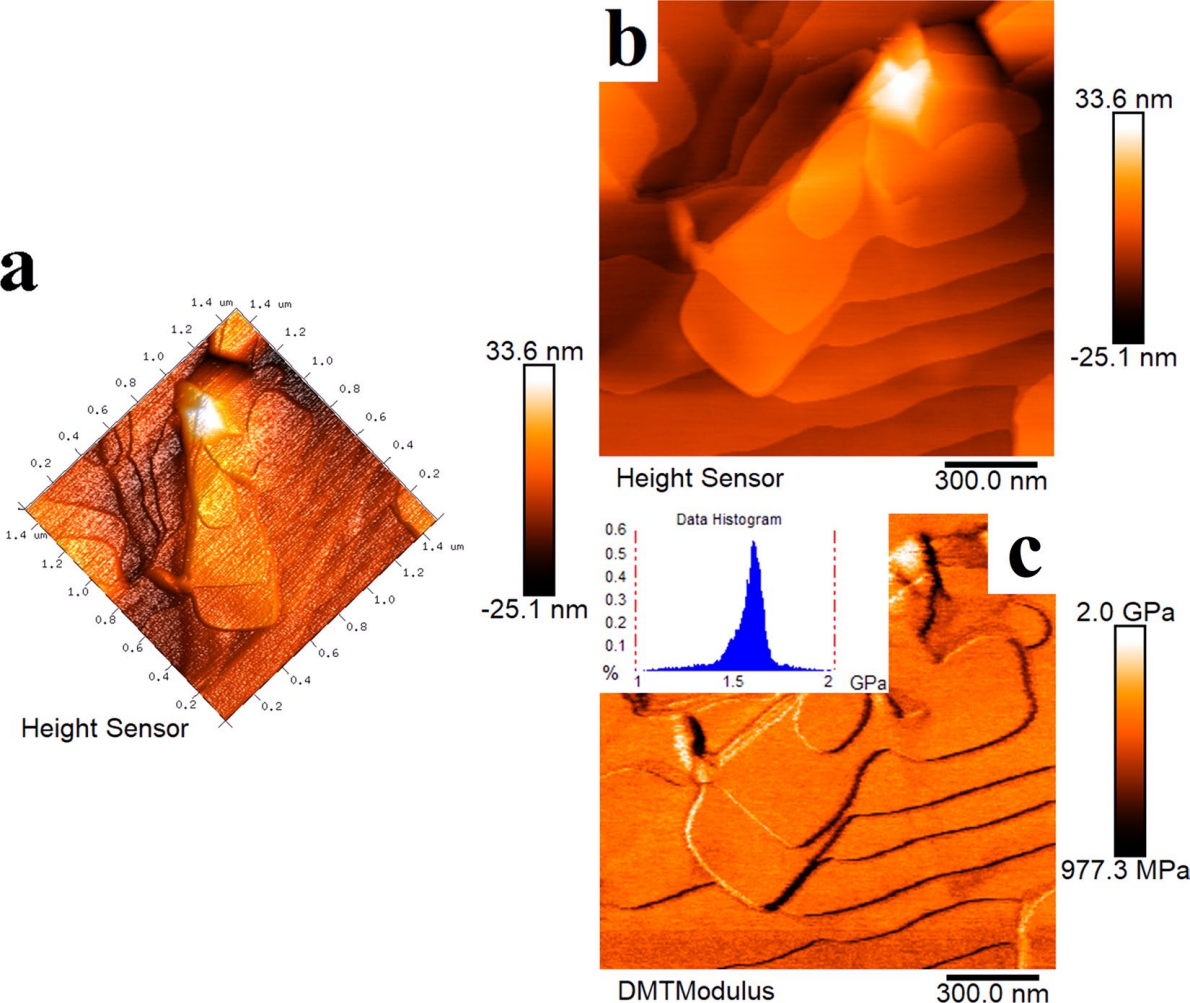
AFM images of BN synthesized at 1100 °C in NaCl-KCl eutectic salts: (**a**) Three-dimensional view; (**b**,**c**) Enlarged plan view.

The morphology of BN synthesized at 1100 °C in NaCl-KCl eutectic salts was further analyzed by AFM. The three-dimensional view and enlarged plan view of BN are shown in Fig. 7a,b. It can be clearly observed that BN has a flake-like structure, which is consistent with the previous SEM and TEM images (Figs 3d and 6a). The flakes are formed in a continuous step shape, the steps do not overlap each other, the surface is flat and smooth, and the width of the BN is approximately 400–450 nm. The elastic modulus of the BN is approximately 1.60 GPa (Fig. 7c).

### FTIR spectrum of r-BN

**Figure 8:**
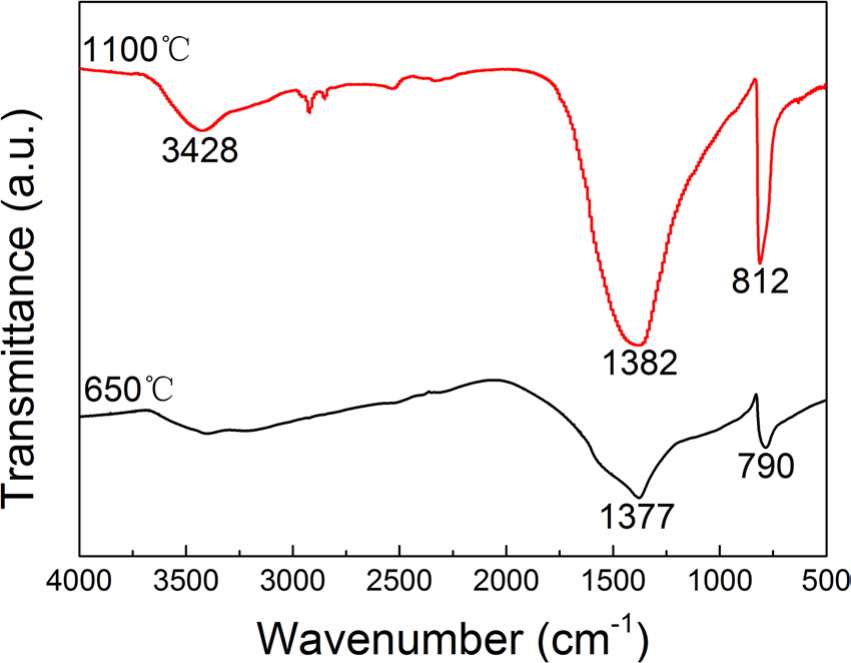
FTIR spectrum of the BN synthesized at 650 °C and 1100 °C in NaCl-KCl eutectic salt.

The FTIR spectrum of the BN synthesized at 650 °C and 1100 °C in NaCl-KCl eutectic salt is shown in Fig. 8. The BN synthesized at 650 °C shows two strong absorption peaks at 1377 cm^−1^ and 790 cm^−1^, which can be referred to as the in-plane B-N stretching vibration and the out-of-plane B-N-B bending vibration, respectively^25^. However, the BN synthesized at 1100 °C forms a weaker and broader absorption peak at 3428 cm^−1^, which may be due to the O-H bond of water adsorbed on the sample surface. The infrared analysis results are consistent with the XRD results, and this further confirms that the resulting product is high-purity r-BN.

### Specific surface area analysis of r-BN

**Figure 9:**
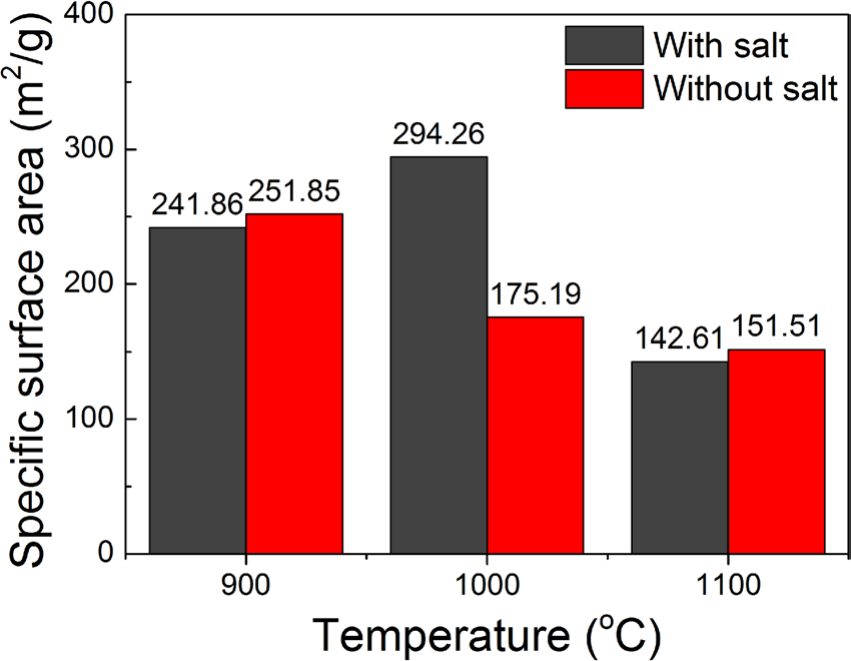
Specific surface area of BN synthesized at different temperatures, with and without salt.

It is well known that the higher the specific surface area of a catalyst carrier, the more favorable the dispersion of the active components, and thus the more conducive to the improvement of its catalytic activity. Therefore, the high specific surface area of BN is of great value as a catalyst carrier^37,38^. With its high specific surface area, BN can be used not only as a catalyst carrier but also as an adsorbent and hydrogen storage material^39–41^. Figure 9 shows the specific surface area of BN synthesized at different temperatures, with and without salt. The synthesis temperature was 900–1100 °C, and the specific surface area of the BN synthesized by MSS^42–49^ method is 142.61–294.26 m^2^/g. The specific surface area of the BN synthesized at 1000 °C is the largest, at 294.26 m^2^/g. This shows that the BN particles produced at this temperature were smaller, as is further confirmed by Fig. 3b–d.

When no salt was added, as the synthesis temperature increased, and the specific surface area of BN, which was in the range of 151.51–251.85 m^2^/g, decreased, indicating that as the temperature rose, the product particles grew. This is further confirmed by Fig. 4b–d. In addition, the two synthesis methods of BN were compared, and the specific surface area of the BN synthesized by the MSS method at 1000 °C was found to be the largest.

### Reaction mechanism of r-BN

**Figure 10:**
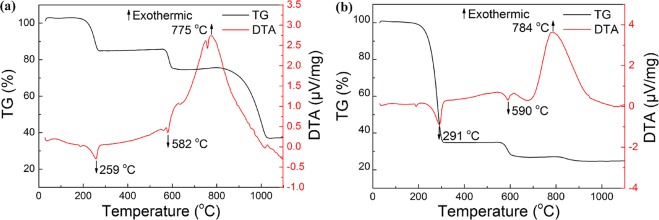
TG-DTA curves of raw materials heated to 1100 °C under nitrogen: (**a**) With salt; (**b**) Without salt.

Figure 10 shows the TG-DTA curves of the raw materials heated from 25 °C at 5 °C/min to 1100 °C under a nitrogen atmosphere. The TG-DTA curves of the raw materials heated to 1100 °C in NaCl-KCl eutectic salts are shown in Fig. 10a. As the heating temperature increased, an endothermic peak appeared at 259 °C with the partial volatilization or decomposition of NH_4_Cl (Eq. 2). With the increase in heating temperature, there was a faint endothermic peak at 582 °C due to the decomposition of KBH_4_ (Eq. 3). During this process, the generated KH and HCl reacted to generate H_2_ (Eq. 4). An exothermic peak appeared at 775 °C, and the mass of the sample decreased with increasing temperature. The reason for this is that the resulting intermediate B_3_N_3_H_6_ (Eq. 5) was slowly dehydrogenated and became exothermic, resulting in the formation of BN (Eq. 6)^50,51^. The total weight loss was approximately 66%.

2 $${{\rm{N}}{\rm{H}}}_{4}{{\rm{C}}{\rm{l}}}_{({\rm{s}})}\to {{\rm{N}}{\rm{H}}}_{3({\rm{g}})}+{{\rm{H}}{\rm{C}}{\rm{l}}}_{({\rm{g}})}$$

$$\Delta {{\rm{G}}}^{{\rm{\theta }}}=97.72-0.27{\rm{T}}\,({\rm{KJ}}\cdot {{\rm{mol}}}^{-1})$$

3 $${{\rm{KBH}}}_{{\rm{4}}({\rm{s}})}\to {{\rm{BH}}}_{{\rm{3}}({\rm{g}})}+{{\rm{KH}}}_{({\rm{g}})}$$

$$\Delta {{\rm{G}}}^{{\rm{\theta }}}=377.28-0.26{\rm{T}}\,({\rm{KJ}}\cdot {{\rm{mol}}}^{-1})$$

4 $${{\rm{KH}}}_{({\rm{g}})}+{{\rm{HCl}}}_{({\rm{g}})}\to {{\rm{KCl}}}_{({\rm{s}})}+{{\rm{H}}}_{{\rm{2}}({\rm{g}})}$$

$$\Delta {{\rm{G}}}^{{\rm{\theta }}}=-\,413.23+0.15{\rm{T}}(\mathrm{KJ}\cdot {{\rm{mol}}}^{-1})$$

5 $$3{{\rm{NH}}}_{{\rm{3}}({\rm{g}})}+3{{\rm{BH}}}_{{\rm{3}}({\rm{g}})}\to {{\rm{B}}}_{{\rm{3}}}{{\rm{N}}}_{{\rm{3}}}{{\rm{H}}}_{{\rm{6}}({\rm{g}})}+6{{\rm{H}}}_{{\rm{2}}({\rm{g}})}$$

$$\Delta {{\rm{G}}}^{{\rm{\theta }}}=-\,642.95-0.02{\rm{T}}\,({\rm{KJ}}\cdot {{\rm{mol}}}^{-1})$$

6 $${{\rm{B}}}_{{\rm{3}}}{{\rm{N}}}_{{\rm{3}}}{{\rm{H}}}_{{\rm{6}}({\rm{g}})}\to 3{{\rm{BN}}}_{({\rm{s}})}+3{{\rm{H}}}_{{\rm{2}}({\rm{g}})}$$

$$\Delta {{\rm{G}}}^{{\rm{\theta }}}=-\,275.26-0.18{\rm{T}}\,({\rm{KJ}}\cdot {{\rm{mol}}}^{-1})$$

Figure 10b shows the TG-DTA curves of the raw materials heated to 1100 °C without adding salt. The quality change in the sample presents in two distinct phases. In the first stage, the temperature range is approximately 200–300 °C and the corresponding mass loss is 64%. In the second stage, the temperature range is 500–700 °C, corresponding to an 8% mass loss. In addition, there is a slight weight loss at 800–900 °C, corresponding to a 2% mass loss. The total weight loss is 74%. Compared with Fig. 10a, the weight loss is quite large, which means that the synthesis of BN using the MSS method can effectively reduce the loss of raw materials during the reaction process.

**Figure 11:**
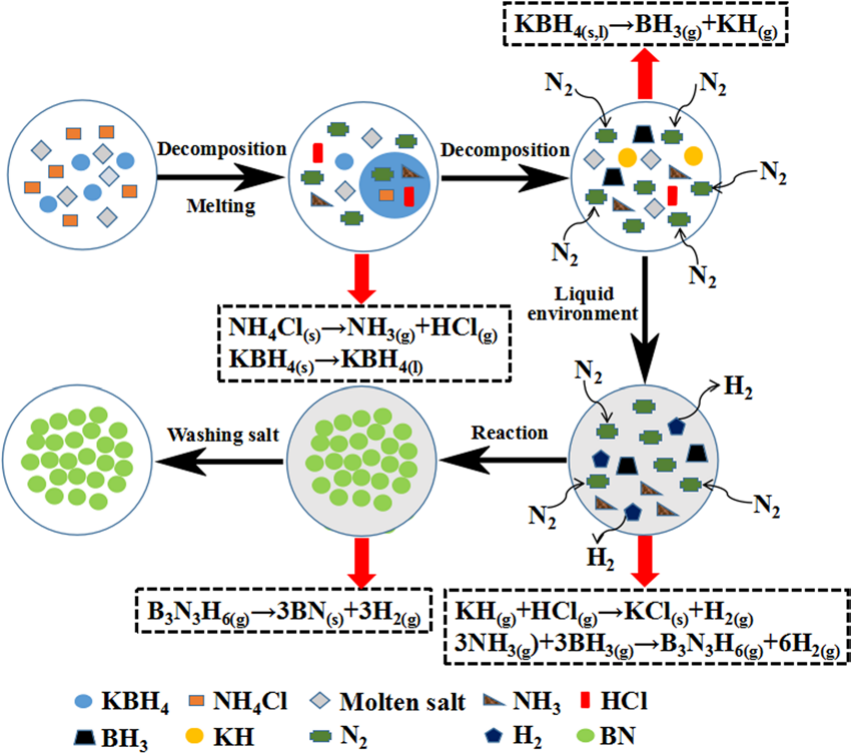
Schematic diagram of the r-BN synthesis by molten salt method.

The reaction mechanism for forming BN was analyzed in conjunction with Fig. 10. A possible formation process for r-BN is shown in Fig. 11. At approximately 200–300 °C, NH_4_Cl is partially volatilized or decomposed into NH_3_. When the temperature is heated to 350 °C, the KBH_4_ begins to melt. Because KBH_4_ is easily decomposed by inorganic acid (e.g., HCl), under the pressure generated by the NH_3_ gas, the KBH_4_ begins to decompose around 580 °C to produce BH_3_. When the heating temperature rises to 657 °C, which is the eutectic melting temperature of NaCl-KCl, the liquid phase environment generated by the molten salt envelopes and penetrates the raw material, preventing the transitional volatilization of NH_4_Cl and slowing down the reaction rate to reduce the loss of generated NH_3_ and BH_3_ with nitrogen. NH_3_ and BH_3_ are further reacted to form gaseous B_3_N_3_H_6_ intermediates, and this is followed by the slow dehydrogenation of B_3_N_3_H_6_ to produce BN nanoparticles. Finally, the BN nanoparticles aggregate into nuclei and grow into flake-like structures along the BN nuclei. The products were washed several times with distilled water to obtain the final product of BN nanoflakes.

### Antioxidant analysis of r-BN

**Figure 12:**
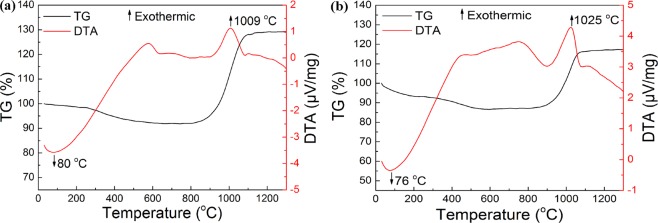
TG-DTA curves of the as-prepared BN nanoflakes in air: (**a**) With salt; (**b**) Without salt.

Studying the antioxidant properties of substances is usually by means of a differential thermal analyzer or the establishment of oxidation kinetics models. In this work, to understand the oxidation resistance of the synthesized BN nanoflakes, the as-prepared BN was subjected to TG and DTA tests. Figure 12 shows the TG-DTA curves of the as-prepared r-BN nanoflakes in air. The TG-DTA curves of the r-BN synthesized at 1100 °C in NaCl-KCl eutectic salts are shown in Fig. 12a. There is an endothermic peak at 80 °C, and the mass loss was due to the volatilization of water molecules in the sample. In addition, a pronounced exothermic peak appears at 1009 °C, which starts at 894 °C and ends at 1084 °C, and the mass of the sample increases and begins to oxidize at 894 °C. Compared with the r-BN triangular nanoplates prepared by Bao *et al*. (BN started to oxidize at 800 °C)^28^, the oxidation resistance increased by approximately 100 °C. This result indicates that the as-prepared BN has excellent oxidation resistance and has good application prospects for high-temperature environments. In addition, when the heating temperature is 800–1200 °C, the BN is oxidized to B_2_O_3_ with an increase of approximately 37% in mass. Theoretically, BN is oxidized to B_2_O_3_ with an increase of approximately 40% in mass, indicating that part of the synthesized BN was not oxidized.

Figure 12b shows the TG-DTA curves of the r-BN synthesized at 1100 °C without adding salt. When the temperature is lower than 600 °C, the sample quality decreases as the heating temperature increased. Furthermore, an exothermic peak appears at 1025 °C, which starts at 901 °C and ends at 1085 °C. When the heating temperature is 800–1200 °C, the BN is oxidized to B_2_O_3_ with an increase of approximately 30% in mass. Compared with the synthesis of BN by MSS, this indirectly shows that the BN particles synthesized without the addition of salt were larger. Moreover, this can be confirmed by Figs 3d and 4d.

## Conclusions

This study shows that the use of the MSS made it easier to synthesize high-purity r-BN nanoflakes with good morphology in a nitrogen atmosphere at low temperatures. KBH_4_ and NH_4_Cl were used as the main raw materials. The effect of salt addition and non-addition on the formation of r-BN was compared, and the heating temperature was also an important factor in the synthesis. High-purity r-BN was synthesized at 650 °C by using the MSS. However, with no salt added, r-BN was synthesized at 900 °C. As the heating temperature increased to 1000 °C, compared with the r-BN synthesized without salt, the r-BN synthesized using the molten NaCl-KCl salt had a uniform morphology. The grain diameter was 200–300 nm and the thickness was 40–70 nm. In addition, at 900–1100 °C, the specific surface area of BN synthesized by the MSS at 1000 °C was the largest, at 294.26 m^2^/g. Furthermore, the BN prepared at 1100 °C had good oxidation resistance in air, and its elastic modulus value was approximately 1.60 GPa.

## Data Availability

For original data, please contact dingjun@wust.edu.cn.

## References

[1] Yin J, Li XM, Zhou JX, Guo WL (2013). Ultralight three-dimensional boron nitride foam with ultralow permittivity and superelasticity. Nano Lett..

[2] Zou CR (2018). Ablation behavior and mechanism of SiO_2f_/SiO_2_, SiO_2f_/BN and Si_3_N_4f_/BN radar wave transparent composites. Corros. Sci..

[3] Zhao YJ, Zhang YJ, Gong HY, Sun HB, Li QS (2014). Gas pressure sintering of BN/Si_3_N_4_, wave-transparent material with Y_2_O_3_-MgO nanopowders addition. Ceram. Int..

[4] Zhi CY (2009). Towards thermoconductive, electrically insulating polymeric composites with boron nitride nanotubes as fillers. Adv. Funct. Mater..

[5] Li Q (2018). Preparation of flake hexagonal BN and its application in electrochemical detection of ascorbic acid, dopamine and uric acid. Sensor. Actuat. B-Chem..

[6] Ashton TS, Moore AL (2017). Foam-like hierarchical hexagonal boron nitride as a non-traditional thermal conductivity enhancer for polymer-based composite materials. Int. J. Heat Mass Tran..

[7] Mandelli D, Leven I, Hod O, Urbakh M (2017). Sliding friction of graphene/hexagonal-boron nitride heterojunctions: a route to robust superlubricity. Sci. Rep..

[8] Katuku K, Koursaris A, Sigalas I (2012). High-temperature stability of polycrystalline cubic boron nitride cutting tool materials in air. Corros. Sci..

[9] Choudhuri I, Pathak B (2018). Ferromagnetism and half-metallicity in a high-band-gap hexagonal boron nitride system. Chemphyschem.

[10] Kamalakar MV, Dankert A, Bergsten J, Ive T, Dash SP (2014). Enhanced tunnel spin injection into graphene using chemical vapor deposited hexagonal boron nitride. Sci. Rep..

[11] Zheng MT, Liu YL, Wang P, Xiao Y (2013). Synthesis and formation mechanism of cubic boron nitride nanorods in lithium bromide molten salt. Mater. Lett..

[12] Melaibari A, Molian P, Shrotriya P (2012). Laser/waterjet heat treatment of polycrystalline cubic/wurtzite boron nitride composite for reaching hardness of polycrystalline diamond. Mater. Lett..

[13] Ye LF (2016). Catalyzed synthesis of rhombohedral boron nitride in sodium chloride molten salt. Ceram. Int..

[14] Zhong B, Zhang XD, Xia L, Yu YL, Wen GW (2017). Large-scale fabrication and utilization of novel hexagonal/turbostratic composite boron nitride nanosheets. Mater. Design.

[15] Shen L, Tan BJ, Willis WS, Suib SL (1994). Characterization of dip-coated boron nitride on silicon carbide fibers. J. Am. Ceram. Soc..

[16] Gautam C (2016). Synthesis and porous h-BN 3D architectures for effective humidity and gas sensors. RSC Adv..

[17] Liang JL (2017). *In-situ* conversion of porous boron nitride to highly crystallized nanoplates-assembled hexagonal boron nitride nanoarchitectures via a metal ion-assisted annealing method. J. Alloy. Compd..

[18] Lin LX, Zheng Y, Zheng Y, Wei KM (2007). Facile synthesis of hexagonal boron nitride fibers and flowers. Mater. Lett..

[19] Torabi O, Golabgir MH, Tajizadegan H, Jamshidi A (2016). Mechanochemical behavior of magnesium-boron oxide-melamine ternary system in the synthesis of h-BN nanopowder. Ceram. Int..

[20] Ye LF (2015). Facile synthesis of hexagonal boron nitride nanoplates via molten-salt-mediated magnesiothermic reduction. Ceram. Int..

[21] Tian Y (2013). Ultrahard nanotwinned cubic boron nitride. Nature.

[22] Solozhenko VL, Kurakevych OO, Godec YL (2012). Creation of nanostuctures by extreme conditions: high-pressure synthesis of ultrahard nanocrystalline cubic boron nitride. Adv. Mater..

[23] Huang YJ, Chen HW, Peng XS, Zhang BT, Chen B (2018). Shock waves preparing cubic boron nitride nanoparticles. J. Alloy. Compd..

[24] Chubarov M, Pedersen H, Högberg H, Henry A (2013). On the effect of silicon in CVD of sp^2^ hybridized boron nitride thin films. Cryst. Eng. Comm..

[25] Chubarov M (2014). Boron nitride: A new photonic material. Physica B.

[26] Chubarov M, Pedersen H, Högberg H, Czigany Z, Henry A (2014). Chemical vapour deposition of epitaxial rhombohedral BN thin films on SiC substrates. Cryst. Eng. Comm..

[27] Oku T, Hiraga K, Matsuda T, Hirai T, Hirabayashi M (2003). Twin structures of rhombohedral and cubic boron nitride prepared by chemical vapor deposition method. Diamond Relat. Mater..

[28] Bao KY (2009). Synthesis of highly crystalline rhombohedral BN triangular nanoplates via a convenient solid state reaction. J. Solid State Chem..

[29] Ding J, Deng CJ, Yuan WJ, Zhu HX, Zhang XJ (2014). Novel synthesis and characterization of silicon carbide nanowires on graphite flakes. Ceram. Int..

[30] Ding J, Zhu HX, Li GQ, Deng CJ, Li J (2014). Growth of SiC nanowires on wooden template surface using molten salt media. Appl. Surf. Sci..

[31] Ding J, Guo D, Deng CJ, Zhu HX, Yu C (2017). Low-temperature synthesis of nanocrystalline ZrC coatings on flake graphite by molten salts. Appl. Surf. Sci..

[32] Chen Y, Deng CJ, Yu C, Ding J, Zhu HX (2018). Molten-salt nitridation synthesis of cubic ZrN nanopowders at low temperature via magnesium thermal reduction. Ceram. Int..

[33] Liu ZL (2019). Molten salt synthesis and characterization of SiC whiskers containing coating on graphite for application in Al_2_O_3_-SiC-C castables. J. Alloy. Compd..

[34] Yang T (2015). Molten salt synthesis of mullite nanowhiskers using different silica sources. Int. J. Min. Met. Mater..

[35] Sun CH, Guo CL, Ma XJ, Xu LQ, Qian YT (2009). A facile route to prepare boron nitride hollow particles at 450 °C. J. Cryst. Growth.

[36] Qu JL, Li Q, Luo C, Cheng J, Hou XM (2018). Characterization of flake boron nitride prepared from the low temperature combustion synthesized precursor and its application for dye adsorption. Coatings.

[37] Wang M (2011). High yield synthesis of novel boron nitride submicro-boxes and their photocatalytic application under visible light irradiation. Catal. Sci. Technol..

[38] Zhou DS (2015). Influence of hexagonal boron nitride on the selective catalytic reduction of NO with NH_3_ over CuOX/TiO_2_. RSC Adv..

[39] Liu ZY (2018). Novel multifunctional cheese-like 3D carbon-BN as a highly efficient adsorbent for water purification. Sci. Rep..

[40] Kumar EM, Sinthika S, Thapa R (2014). First principles guide to tune h-BN nanostructures as superior light–element–based hydrogen storage materials: role of the bond exchange spillover mechanism. J. Mater. Chem. A.

[41] Li Q (2016). Porous hexagonal boron nitride whiskers fabricated at low temperature for effective removal of organic pollutants from water. Ceram. Int..

[42] Ding J, Deng CJ, Yuan WJ, Zhu HX, Li J (2013). The synthesis of titanium nitride whiskers on the surface of graphite by molten salt media. Ceram. Int..

[43] Ding J (2015). Preparation and characterisation of porous biomorphic SiC/C ceramic from molten salt. Ceram. Int..

[44] Ding J, Zhu HX, Li GQ, Deng CJ, Chai ZN (2016). Catalyst-assisted synthesis of α-Si_3_N_4_ in molten salt. Ceram. Int..

[45] Chai ZN (2016). Ni-catalyzed synthesis of hexagonal plate–like alpha silicon nitride from nitridation of Si powder in molten salt media. Adv. Powder Technol..

[46] Kan XQ (2017). Low-temperature fabrication of porous ZrC/C composite material from molten salts. Ceram. Int..

[47] Kan XQ, Ding J, Zhu HX, Deng CJ, Yu C (2017). Low temperature synthesis of nanoscale titanium nitride via molten-salt-mediated magnesiothermic reduction. Powder Technol..

[48] Liu HX (2019). Low-temperature synthesis and properties of VN nanopowder via a combined molten salt nitridation and magnesium thermal reduction. Ceram. Int..

[49] Wang X (2019). Preparation and application of ZrC-coated flake graphite for Al_2_O_3_-C refractories. J. Alloy. Compd..

[50] Hu JQ (1999). Synthesis and characterization of nanocrystalline boron nitride. J. Solid State Chem..

[51] Bi JQ (2009). Large-scale synthesis of BN nanotubes using carbon nanotubes as template. Mater. Lett..

